# Predicting Genome Architecture: Challenges and Solutions

**DOI:** 10.3389/fgene.2020.617202

**Published:** 2021-01-22

**Authors:** Polina Belokopytova, Veniamin Fishman

**Affiliations:** ^1^Natural Sciences Department, Novosibirsk State University, Novosibirsk, Russia; ^2^Institute of Cytology and Genetics Siberian Branch of Russian Academy of Sciences (SB RAS), Novosibirsk, Russia

**Keywords:** Hi-C, modeling, polymer physics, machine learning, predicting approaches

## Abstract

Genome architecture plays a pivotal role in gene regulation. The use of high-throughput methods for chromatin profiling and 3-D interaction mapping provide rich experimental data sets describing genome organization and dynamics. These data challenge development of new models and algorithms connecting genome architecture with epigenetic marks. In this review, we describe how chromatin architecture could be reconstructed from epigenetic data using biophysical or statistical approaches. We discuss the applicability and limitations of these methods for understanding the mechanisms of chromatin organization. We also highlight the emergence of new predictive approaches for scoring effects of structural variations in human cells.

## Studying Genome Architecture: Methods and Mechanisms

The human genome has a three-dimensional structure, which folds in the nucleus, producing specific chromatin interactions. These chromatin interactions can be experimentally assessed by modern microscopy methods (reviewed in Boettiger and Murphy, 2020) or sequencing approaches, such as genome-wide modifications of chromatin conformation capture (Hi-C) (Lieberman-Aiden et al., 2009; Rao et al., 2014), split-pool recognition of interactions by tag extension (Quinodoz et al., 2018), and genome architecture mapping (Beagrie et al., 2017). These methods are covered by comprehensive reviews (Kempfer and Pombo, 2020) and comparative studies (Fiorillo et al., 2020). Here, we focus mainly on the Hi-C technique and its results because this method was most widely applied in various genomic studies during the last decade, allowing the accumulation of a huge amount of experimental data. Both methodological aspects of the Hi-C technique (Fiorillo et al., 2020) and biological principles revealed by applying this method to study genome architecture (Szabo et al., 2019) are discussed in detail in several recent reviews. We refer readers to Box 1, where we briefly discuss the main concepts of this field for the sake of completeness.

**BOX 1 Start of Box 1 Hi-C technology uncovers principles of genome organization.**

Hi-C includes crosslinking and digestion of chromatin, followed by proximity ligation and sequencing of ligation products (Lieberman-Aiden et al., 2009; Rao et al., 2014). During the proximity ligation step, only those genomic regions that spatially co-localize have a chance to be ligated. Thus, counting ligation products by next-generation sequencing allows deciphering the spatial proximity of loci. Although several single-cell Hi-C methods are published (Flyamer et al., 2017), the technique is most often applied to large cell populations, and ligation event frequency (also referred to as *interaction* or *contact* frequency throughout this review) should be interpreted as the average frequency of loci co-localization among the studied cell population. This snapshot of averaged chromatin contacts in a population, typically represented by a matrix of pairwise interaction frequencies, is known as a Hi-C map.

Using Hi-C and other methods, several important principles of genome architecture were recently discovered. At the largest scales, chromosomes occupy distinct territories, showing only limited intermingling (Tavares-Cadete et al., 2020) and characterized by an exponential decay of contact frequencies with the genomic distance between loci (Lieberman-Aiden et al., 2009). Within the territories, one can distinguish compartments that correspond to different chromatin types (Lieberman-Aiden et al., 2009). Mechanisms underlying compartment formation are actively debated, and there is a growing body of theoretical and experimental pieces of evidence suggesting the essential role of liquid–liquid phase separation in these processes (Kantidze and Razin, 2020; Razin and Gavrilov, 2020; Razin and Ulianov, 2020). At a finer scale, specific loci may preferentially interact with each other, forming topologically associated domains (TADs) (Dixon et al., 2012), stripes (Vian et al., 2018), cliques (Petrovic et al., 2019), and loops (Rao et al., 2014). Although the terminology is not well established in this field (de Wit, 2020), the current mechanisms underlying the formation of these structures fall into two categories.

First is a recently proposed loop extrusion mechanism (Sanborn et al., 2015; Fudenberg et al., 2016). It is considered that ring-shaped cohesin and condensin proteins bind chromatin and form and continuously extend loops in an ATP-dependent manner. Extrusion stops encountering another extrusion complex or, in the case of cohesins, when reaching CTCF protein bound to DNA in a specific orientation. This results in increased interaction frequency between loci bound by cohesin, displayed on Hi-C maps as loops (two-point interactions) (Rao et al., 2014) or stripes (one-to-many-points interactions) (Vian et al., 2018). The chromatin interaction patterns arising from loop extrusion mechanisms could be qualitatively described by the landscape of CTCF binding and also depend on the loading and processivity of cohesin (Fudenberg et al., 2016). Moreover, loop extrusion results in increased proximity of all loci located between convergently oriented CTCF sites, which is captured by the formation of looping domains (Rao et al., 2014).

The second mechanism responsible for the formation of loops and cliques is mediated by the formation of regulatory protein complexes, for example, polycomb complexes (Eagen et al., 2017), and certain transcription factors (Petrovic et al., 2019). This mechanism is at least partially independent of cohesin-mediated extrusion because the subset of loops remains stable upon degradation of the cohesin complex (Rao et al., 2017).

It is important to note that profiles of chromatin interactions captured by the Hi-C experiment are formed by the joint action of different mechanisms. For example, the formation of TADs, which represent self-interacting regions in the genome, is affected both by loop extrusion and compartmentalization processes (Szabo et al., 2019; de Wit, 2020), which is consistent with both convergent CTCF sites and chromatin state transition enrichment at TAD boundaries (Dixon et al., 2012; Rao et al., 2014; Huang et al., 2015).

## Why Modeling 3-D Genome Folding?

The models and algorithms predicting genome architecture can be used in different ways. First, we can apply modeling to get new insights or test our hypotheses of molecular mechanisms underlying 3-D genome folding. Polymer modeling is used more often for this purpose, but convolutional neural networks, such as, for example, Akita (Fudenberg et al., 2020) and DeepC (Schwessinger et al., 2020), also enable identifying the main chromosome features contributing to genome architecture. Such approaches give remarkable results. During the last few years, we gained a significant amount of data describing the main features of 3-D genome folding and understanding the molecular mechanisms underlying these data, including loop extrusion and phase separation, which was largely facilitated by biophysical modeling and statistical analysis of chromatin properties. This field of research is well described in reviews (Imakaev et al., 2015; Lin et al., 2019). However, known mechanisms do not explain all 3-D chromatin features, which limits hypothesis-driven models and further research is required to explain them.

Second, 3-D genome models can be used to predict functional consequences caused by changes in 3-D genome folding. It is shown that alterations of chromatin topology accompanying genomic variations, especially large structural variations, can cause changes of gene expression (Franke et al., 2016; Rodríguez-Carballo et al., 2017; Kraft et al., 2019). One can find examples of such gene expression changes and their underlying mechanisms in the last part of this review. In these cases, modeling of 3-D genome architecture is essential for accurate prediction of the consequences of the genomic mutations.

Last, one can use modeling for predicting the 3-D genome architecture of new data. It is possible to predict chromatin interactions for different cell types lacking experimental Hi-C data (Belokopytova et al., 2020). Machine learning methods often gain applicability in this way.

## Which 3-D Genome Structures Can Be Predicted, and Why They Are Relevant?

Chromosome-capturing methods, such as Hi-C, allow deciphering the main features of chromatin folding. Since the first Hi-C experiments, chromatin structures as compartments, TADs, and loops were revealed (see Box 1 for details of mechanisms underlying these structures). In the following, we describe the main Hi-C map features and algorithms used to predict them. Also, it may be helpful for readers new to the field to use the table of algorithms (Table 1) containing algorithms for predicting different 3-D genome features.

**TABLE 1:** Tools for modeling and predicting chromatin interactions.

**Tool name**	**Input features**	**Target features**	**Method/algorithm**
See review by Xu et al. (2018)	Histone marks, TFs binding, DHS	Promoter–enhancer interactions	See review by Xu et al. (2018)
MacPherson et al. (2018) model	HP1, H3K9me3	Compartments	Polymer modeling
MichroM + MEGABASE (Di Pierro et al.)	Histone marks, TFs binding	Compartments	NN classifier + polymer modeling
Huang et al. (2015) model	Histone marks	TADs	BART
3Disease Browser (Li et al., 2016)	Enhancers and TAD boundaries	Rearranged TADs	Linear model
Lollipop (Kai et al., 2018)	Chip-seq data, CTCF directionality	Loops	ML ensemble classifier (random forest)
3DEpiloop (Al Bkhetan and Plewczynski, 2018)	Histone marks, TFs binding	Loops	ML ensemble classifier (random forest)
CTCF-MP (Zhang et al., 2018)	CTCF binding, DHS, nucleotide sequence	Loops	ML ensemble classifier/NN (Boosted trees/word2vec)
EpiTensor (Zhu et al., 2016)	Histone marks, TFs binding	Loops	Tensor modeling + PCA
DeepMILO (Trieu et al., 2020)	Sequence of loop anchors	Rearranged loops	CNN and RNN
3D-GNOME (Sadowski et al., 2019)	CTCF ChIA-PET	Rearranged loops	linear models
3DPredictor (Belokopytova et al., 2020)	CTCF, RNA-seq	Whole hi-c map	ML ensemble regression (gradient boosting)
Hi-C Reg (Zhang et al., 2019)	Histone marks, TFs binding, DHS	Whole hi-c map	ML ensemble regression (random forest)
Akita (Fudenberg et al., 2020)	Sequence	Whole hi-c map	CNN
DeepC (Schwessinger et al., 2020)	Sequence	Whole hi-c map	CNN
Yifeng Qi and Bin Zhang model (Qi and Zhang, 2019)	CTCF binding, Chromatin states	Whole hi-c map	Polymer modeling
HiP-HoP (Buckle et al., 2018)	CTCF and cohesin binding, Histone marks or DHS	Whole hi-c map	Polymer modeling
Rowley et al. (2017) model	GRO-seq + CTCF binding	Whole hi-c map	Explicit algebraic model
PRISMR (Bianco et al., 2018)	Wild-type Hi-C data	Whole hi-c map in mutated cells	Polymer modeling

*DHS, DNAse I hypersensitivity sites; TFs, transcription factors; TADs, topologically associated domains; ML, machine learning; NN, neural network; CNN, convolutional neural network; RNN, recurrent neural network; BART, Bayesian additive regression trees; PCA, principle component analysis.*

### Promoter–Enhancer Interactions

Interactions between promoters and enhancers are essential for expression regulation. Pioneering attempts to find such regulatory connections rely on either the correlation of epigenetic marks of promoters and enhancers across different cell types or evolutionary conservation of promoter–enhancer proximity in the linear DNA molecule (Spicuglia and Vanhille, 2012; Andersson and Sandelin, 2020). With the advent of genome-wide 3C-methods, we gain the ability to measure spatial proximity between genomic segments. The question about the exact role of spatial contacts between regulatory elements in the control of gene expression is still under active debate; however, much research defines “interacting” enhancers and promoters as pairs of loci belonging to the anchors of one Hi-C loop. Although we argue that using this loop-based definition of interacting promoters and enhancers might be confusing (see Box 1 and limitations section below for additional discussion), several algorithms are designed to predict enhancer–promoter pairs located within the anchors of one loop (Whalen et al., 2016).

### Loops

Instead of predicting whether promoters and enhancers overlap loop anchors, some algorithms, such as Lollipop (Kai et al., 2018), 3DEpiloop (Al Bkhetan and Plewczynski, 2018), and EpiTensor (Zhu et al., 2016), are designed to directly infer all loop positions using epigenetic data. In mammals, most of the looping interactions are formed due to the cohesin-mediated loop extrusion process (see Box 1 for details). Thus, some algorithms, such as CTCF-MP (Zhang et al., 2018) or Lollipop (Kai et al., 2018), are focused exclusively on the prediction of CTCF-mediated interactions or separately access quality of prediction for CTCF-mediated and all other loops as in the DeepMILO algorithm (Trieu et al., 2020).

### TADs

TADs have the shape of triangles on Hi-C maps, which indicates an increase of chromatin interaction frequency within TADs and insulation at TAD borders. These structures are largely dependent on the extrusion process and also influenced by other mechanisms (see references provided in Box 1 for discussion of the TAD definition and current views on mechanisms explaining TAD formation). TADs are also relevant for promoter–enhancer interactions as the majority of the functional interactions occur within the same TAD. It is known that TAD boundaries are enriched by CTCF binding sites (usually in convergent orientation) and different epigenetic marks (Dixon et al., 2012). Based on these observations, Huang et al. (2015) use ChIP-seq data for different proteins in a computational model predicting TAD boundaries and chromatin interaction hubs.

### Compartments

Chromatin compartments are the main features of distant contacts revealed by chromosome conformation capture. Hi-C maps show that interactions occur more often within each compartment rather than across compartments (Lieberman-Aiden et al., 2009). The presence of compartments results in a checkerboard-like (or “plaid-like”) pattern of contacts on Hi-C maps. It is shown that compartments reflect the clustering of different types of chromatin (see Box 1 for details). Seminal work proposed binary division of the genome into eu- and heterochromatin, which correspond to A- and B-compartments. Subsequent research extends this view, suggesting that multiple chromatin states exist, each described by a unique profile of spatial interactions (Rao et al., 2014). In accord with this, several models are proposed, allowing the prediction of compartmental interactions based on epigenetic data (Di Pierro et al., 2017; MacPherson et al., 2018). Most of these algorithms utilize physical modeling to infer spatial chromatin interactions. Machine learning methods are often used as a part of the algorithm to attribute genomic loci to a certain compartment based on its epigenetic signatures.

### Hi-C Maps

Predictions of all aforementioned features require similar epigenetic information. Thus, it should be possible to develop an algorithm predicting all topological structures simultaneously. Because it is widely assumed that biologically relevant interactions do not occur at a distance above several megabases, most of the algorithms limit their prediction to these distances, which reduces computational time and resources. For instance, machine learning algorithms, such as 3Dpredictor (Belokopytova et al., 2020), HiC-Reg (Zhang et al., 2019), Akita (Fudenberg et al., 2020), and DeepC (Schwessinger et al., 2020), predict all interactions within an ∼1–3 Mb window. In addition, some polymer modeling approaches, such as Hip-Hop (Buckle et al., 2018) and PRISMR (Bianco et al., 2018), could be used to predict the whole Hi-C heat map.

### From Contact Frequencies to 3-D Models

Hi-C and other 3C-based methods provide a snapshot of pairwise interactions between loci. Although we call this “3-D” information, it cannot be trivially transformed into 3-D structures. An approach known as restraint-based (RB) modeling interprets the 3C-based data as a set of spatial restraints to build a 3-D model of the chromatin fiber by satisfying the input restraints. The chromatin fiber is represented as a polymer of consecutive monomers, and several computational optimization strategies can be employed to find 3-D models of chromatin (Dekker et al., 2013; Serra et al., 2015). The challenge of predicting 3-D genomic structures from high-resolution chromosome conformation capture data was recently taken by several groups, and we refer the reader to the recent review by Kimberly MacKay and Anthony Kusalik describing problems and solutions in this field (MacKay and Kusalik, 2020) and to the articles collected in the recently published book *Modeling the 3D Conformation of Genomes* (Tiana and Luca, 2019).

## How Do the Modeling Algorithms Work? Problems and Limitations

All models and algorithms that are currently used to infer chromatin contacts from epigenetic data could be divided into two categories. First are the models derived from the physical simulation of chromatin behavior, i.e., polymer modeling. The second includes statistical algorithms searching for interdependencies between genetic and epigenetic properties and patterns of 3-D contacts. Here, we described the principles and limitations of both approaches.

## Polymer Modeling

The physics of chromatin has been the subject of intense research over many decades. Seminal studies by de Gennes and Witten (1980) provide basic rules describing polymer behavior under different conditions. Importantly, these studies show that, when a polymer is large (i.e., its size increases the size of individual monomers significantly), its physical properties do not depend on the monomer’s chemical structure. Instead, the behavior of a polymer depends on several physical parameters, such as monomer concentration, solvent quality, and temperature. For different combinations of these parameters, the polymer would exist in one of the well-described equilibrium states, such as the random coil, the swollen coil, the equilibrium globular state, and others (Fudenberg and Mirny, 2012). Thus, knowing the key parameters and using the laws of polymer physics would allow the description (and prediction) of chromatin behavior within the nucleus. These ideas gave rise to the first physical models of chromatin architecture.

**FIGURE 1:**
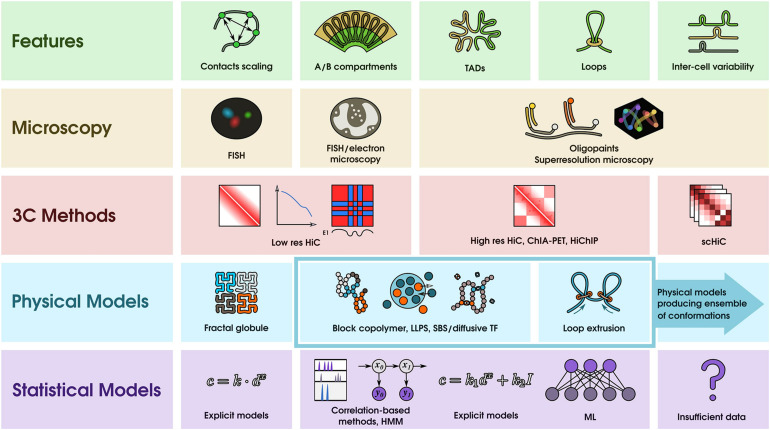
Modeling and predicting the main features of the 3-D-genome organization using physical and statistical approaches. The features row contains a schematic representation of the main features of 3-D-genome organization: scaling of contacts with genomic distance, compartments, TADs, loops, and inter-cell variability of genome architecture (from left to right). The 3C methods row shows that contact scaling and compartments could be found using low-resolution Hi-C data, whereas identification of loops and dissection of TAD structure requires high resolution. Studying inter-cell variability is challenging and could be done using single-cell Hi-C approaches (scHi-C). Microscopy methods shown in the second row include conventional 3D-FISH (fluorescent *in situ* hybridization) to measure spatial distances; electron microscopy, which is helpful to visualize segregation of eu- and heterochromatin; and modern super-resolution microscopy methods, which, in combination with oligopaints, allow dissection of the internal structure of TADs in individual cells (Boettiger and Murphy, 2020; Szabo et al., 2020). Physical description of chromatin interactions (fourth row) includes generic models such as the fractal globule as well as locus-specific models. For the latter, researchers employ block-copolymer models and models with chromatin binders, such as strings and binders switch (SBS) and diffusive transcription factor (TF) models and concepts of liquid–liquid phase separation (LLPS). All these physical models allow studying the dynamics and inter-cell variability of 3-D structures, providing ensembles of possible chromatin conformations (this is schematically shown in the last cell of the physical models row). Statistical methods (the last row) could utilize interconnections between epigenetic data and chromatin organization using different approaches. This includes approaches in which explicitly defined algebraic expressions contain free parameters, which could be fit from the data, hidden Markov models (HMM), and various machine learning (ML) algorithms. TADs, loops, and compartments were predicted using these methods. However, for single-cell data, these approaches are not applicable, mainly due to the large amount of data required for the implementation of these algorithms.

Development and validation of physical models during recent decades are linked to the development of experimental techniques measuring genome architecture (Figure 1). The presence of chromosome territories as well as measures of mean distances between defined loci by FISH disagree with basic swollen coil or random coil polymer properties (Hahnfeldt et al., 1993). There were multiple attempts to improve these disagreements, of which the fractal globule (Mirny, 2011) is currently the most accepted. This model, originally proposed by Grosberg et al. (1988) suggests that chromatin exists in a highly unknotted fractal-like non-equilibrium state, and the predictions obtained using this model fit well with the experimentally measured scaling of Hi-C contacts (Lieberman-Aiden et al., 2009).

Although the fractal globule recapitulates the experimentally observed scaling of chromatin contacts better than the equilibrium globule state, it is still far from a complete description of chromatin folding in a real cell. Not to mention all disagreements (see Grosberg, 2016, for a detailed review), the fractal globule represents a pictorial description of the chromatin structures and does not include locus-specific features. Thus, to build a more comprehensive description of chromatin conformation and dynamics in a real cell, active (energy-consuming) locus-specific mechanisms should be introduced into the system.

One such mechanism, which maintains the structure of chromatin, is a loop extrusion process (see Box 1 for details on this mechanism). This process was recently introduced into physical models of chromatin by Fudenberg et al. (2016) and Sanborn et al. (2015), and later experimentally validated by Ganji et al. (2018), Davidson et al. (2019), and Kim et al. (2019). A recent preprint from Banigan et al. (2020) shows another impressive application of polymer modeling in which it helps to investigate if a one- or two-sided loop extrusion model works in the cell and to identify a class of one-sided extrusion models that can reproduce *in vivo* experiments. The models of loop extrusion show good agreement with the experimental Hi-C data. Importantly, loop extrusion models use epigenetic information about CTCF binding to account for CTCF-mediated extrusion barriers. This allows making the model locus-specific; moreover, modifying CTCF anchors *in silico* results in different chromatin packaging as revealed by the models (Sanborn et al., 2015). Thus, such physical models allow predicting chromatin packaging and its perturbations knowing CTCF-binding sites.

Another class of locus-specific models is designed to study and predict the packaging of different chromatin types. Distinct types of chromatin differentially interact with themselves and surrounding proteins. This can be imagined as a polymer composed of several distinct units or blocks. Such polymers are called block copolymers, and their behavior could be modeled knowing the interaction potential between blocks (Bates and Fredrickson, 1990). Several attempts have been made to apply this logic for modeling chromatin interactions in *Drosophila* and *Human* (Jost et al., 2014; Di Pierro et al., 2016; Ulianov et al., 2016). These models predict that specific preferences of interactions between similar blocks of chromatin result in spatial segregation of distinct chromatin domains in the process of liquid–liquid phase separation (Nuebler et al., 2018).

Block copolymer models rely on the epigenetic information about histone modifications and/or architectural factor binding to assign DNA segments to specific chromatin types. Once developed, these models could be used to predict chromatin architecture if epigenetic data is available. Indeed, several studies show that such prediction recapitulates Hi-C data very well (Di Pierro et al., 2017), especially when accounting for the loop extrusion process (Nuebler et al., 2018; Qi and Zhang, 2019).

To further extend block copolymer models, one should consider the physical nature of interactions between blocks. In a nucleus, these interactions are mediated by specific factors, such as polycomb-group proteins (Plys et al., 2019; Eeftens et al., 2020), BRD-domain containing proteins (Gibson et al., 2019), HP1 (Larson et al., 2017; Sanulli et al., 2019), mediator and RNA polymerase II (Cho et al., 2018), or interactions between DNA and nuclear lamina proteins (Chiang et al., 2019; Ulianov et al., 2019). The above-described block copolymer models account for these interactions implicitly by setting specific interaction potentials between different block types. Other models explicitly introduce binder proteins that mediate interactions in the system.

There are multiple ligand-binding theories applied to model DNA–protein interactions in chromatin, reviewed, for example, in Teif and Rippe, 2010. Among recent models that aim to explain genome-wide interaction profiles revealed by 3C-based methods, several consider specific chromatin binders, such as HP1 (Teif et al., 2015; MacPherson et al., 2018), lamina proteins (Chiang et al., 2019; Ulianov et al., 2019), or generic active and inactive complexes (Brackley et al., 2016b), whereas others describe binders, such as abstract molecules with defined physical properties but unknown biological nature (Nicodemi and Prisco, 2009; Barbieri et al., 2012; Brackley et al., 2013, 2017; Chiariello et al., 2016). Mechanistically, chromatin clustering may be reproduced by these models either due to the affinity of binders or because of multivalent interactions between binders and chromatin, which results in bridging-induced attraction (Brackley et al., 2013, 2017; Johnson et al., 2015). In addition to compartmentalization, these mechanisms could explain TAD and loops formation (Brackley et al., 2016b). For more details on these and other physical models, we refer the reader to a recently published extensive review (Brackey et al., 2020) and a collection of articles provided with the book (Tiana and Luca, 2019).

Here, it is pertinent to note that the binder positions are inferred from epigenetic data even in those models that use “abstract” binders. This allows predicting chromatin folding in normal and mutated genomes, knowing epigenetic data with high accuracy (Scialdone et al., 2011; Bianco et al., 2012, 2018; Brackley et al., 2016a,b; Barbieri et al., 2017; Chiariello et al., 2017; Kragesteen et al., 2018). For example, the Hip-Hop model (Buckle et al., 2018) infers binder positions based on H3K27 acetylation data and/or chromatin accessibility, and the authors show that this epigenetic information is sufficient for prediction of chromatin interactions. In the PRISMR model (Bianco et al., 2018), Hi-C data obtained from wild-type cells are used to define the number of binder types and their affinities, and this information can be further used to model chromatin conformation after a deletion or duplication event occurs.

The examples mentioned above show that physical modeling could be a powerful tool for both validation of proposed molecular mechanisms underlying chromatin architecture and predicting spatial interactions based on epigenetic data. In the following, we discuss some limitations that should be addressed to allow a comprehensive description of genome organization by physical modeling.

### Limitations of Physical Models

#### Physical Modeling Is Hypothesis-Driven

As was mentioned above, physical models rely on an explicitly defined set of rules to describe polymer behavior. However, we are still far from a complete understanding of all biophysical processes involved in chromatin organization. Thus, it is clear that none of the currently developed models can accurately explain all details of genome architecture and dynamics.

For example, PRISMR and Hip-Hop models introduce specific binders whose positions and affinity could be inferred from experimental Hi-C or ChIP-seq data. The problem is not only that we do not know the correspondence between the model’s abstract binders and real proteins. The major concern is that these abstract binders might not be given the same physical properties as real proteins. Biochemical dissection of regulatory complexes, such as PRC1 or Mediator, show the complexity of their structural organization and regulation, which is not described by current models. This limits modeling approaches to qualitative predictions of trends rather than quantitative comparison with contact maps.

#### Inferring Key Physical Parameters Might Be Challenging

There are many biophysical parameters that are currently unknown but essential for modeling. This includes affinity constants and concentrations of chromatin binders, the position of boundaries, and processivity of loop extruders and other factors. One solution to this problem is extracting the missing parameters from available ChIP-seq data. For example, in the MEGABASE + MiChroM model developed by Di Pierro and colleagues, chromatin states are first inferred from epigenetic data using a machine learning approach and then used in a block copolymer model optimized to fit Hi-C data (Di Pierro et al., 2017). However, in many cases, available ChIP-seq data is only indirectly connected to the affinity and concentration of the key architectural factors, and the dependence between ChIP-seq signals and biophysical properties of chromatin may vary in different cell types. Thus, the model developed using one cell type might not be well transferable to another.

There are also models that fit their parameters directly using Hi-C data. This is, for example, the PRISMR model (Bianco et al., 2018), which defines binder types and positions based on Hi-C maps. The transferability of this model to other cell types or loci without knowing corresponding experimental Hi-C data could be problematic.

There are also several technical parameters of simulation that could influence the results, including the finite volume effect, polymer conformation used for model initialization, equilibration time, sampling size, etc. We refer those readers interested in this subject to a recent review describing potential pitfalls and methods developed to overcome these limitations (Gartner and Jayaraman, 2019).

#### Physical Modeling Is Computationally Intensive and Often Requires Coarse-Graining

Using a polymer modeling approach is computationally intensive. Technically, the vast majority of the physical models describe chromatin as a string with beads. Ideally, each bead should represent a single nucleosome as histone octamers are monomers of chromatin organization. However, this leads to a huge number of beads required to simulate chromosome-scaled loci. The behavior of beads is typically simulated using LAMMPS software, which is computationally intensive for such a large number of objects. Great computational resources are needed for every modeling attempt, and these are not always accessible. Although it is possible to model only a particular chromosomal region, whole chromosome or whole genome modeling is computationally too expensive.

One solution could be to decrease the resolution and use more coarse-grained models, with which several atoms or molecules are grouped and represented by a single simple object. However, this comes at a cost of the inability to resolve fine patterns of interactions. There are multiple levels of chromatin coarse-graining, starting from atomic resolution and up to hundreds of thousands of base pairs, each suitable for the specific problem of interest (see Table 1 in the recent review published by Brackey et al., 2020). The choice of coarse-graining should be considered carefully in order to find a balance between the detail of the model and computational cost.

To sum up, physical modeling is essential for validating hypotheses about mechanisms driving chromatin organization. When using epigenetic data to infer properties of chromatin monomers, it is easy to repurpose a physical model from hypothesis validation to prediction of locus-specific chromatin organization. However, there are several limitations of these predictions, and we next describe another class of approaches based on machine learning techniques that have the potential to overcome some of the aforementioned limitations.

## Statistical Approach

It is known that different epigenetic marks and transcriptional factors correlate with various regulatory elements, chromatin states, and other genomic features. For example, histone modification H3K9me3 correlates well with constitutive heterochromatin, which correlates with the B compartment (Strom et al., 2017), TAD boundaries are enriched by CTCF protein (Dixon et al., 2012; Rao et al., 2014), and open chromatin regions are enriched by specific histone modification. Thus, one can simply use regression to predict 3-D genome features based on epigenetics data. For example, correlation-based methods are used for the prediction of enhancer–promoter interactions using histone modifications, CAGE, ChIP-seq, and other chromatin features as input (Xu et al., 2020).

Although linear models could explain 3-D organization to some extent, it is clear that certain dependencies between genetic features and chromatin interactions are not linear. The most prominent example of such non-linearity is the scaling of the average chromatin contact frequency with genomic distance, which could be well described as a power law. This dependence, P(s) ∼ s^x, has only one free parameter x, which could be easily obtained by fitting experimental data. Of course, it is not enough to account for distance dependence to obtain accurate estimations of contact frequencies. One should also describe locus-specific insulation, compartmentalization, and other features of genome organization. This description should be done in the form of algebraic expressions with some free parameters that could be fit from the data. This was utilized recently by Rowley et al. (2017), who proposed an algebraic expression combining linear and exponential terms to predict genomic contacts based on GRO-seq transcription data, CTCF binding, and genomic distance. As a result, Rowley et al. simulate Hi-C maps including main 3-D structures, such as TADs and loops with high accuracy.

**FIGURE 2:**
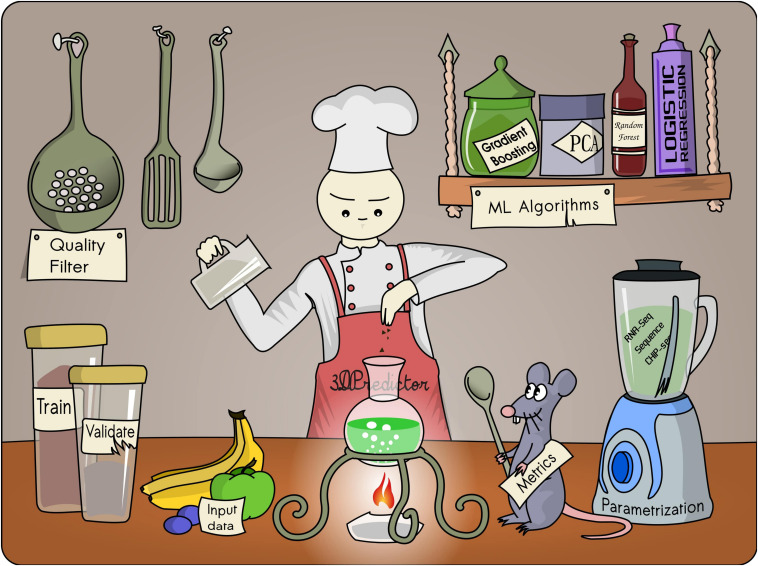
In designing a machine learning–based algorithm, one should carefully choose the main “ingredients” required for good prediction.

However, there might be multiple non-linear dependencies between histone modifications, transcription factor binding, and chromatin interactions, which cannot be defined analytically as an algebraic expression, such as a power law. These dependencies could be found by sophisticated machine learning algorithms, such as logistic regression, gradient boosting, random forest regression, neural networks, and others (Eraslan et al., 2019; Figure 2).

Machine learning algorithms operate with a numerical representation of input information (*features*): nucleotide sequence; genomic distance or epigenetic marks; and experimentally measured target feature values, such as contact frequency between loci, positions of loop anchors, etc. The main result of machine learning training is a function that transforms input features into predictions of target values. The similarity between predictions and experimental data is measured using a user-defined loss function. During a training step, the portion of available data called the training subsample is used to optimize the transforming function so that the loss function is minimal; this is how the algorithm finds interdependencies between features and target values. These interdependencies might represent general biological mechanisms or be subsampling artifacts specific to the training subsample. Moreover, the function transforming the input features into predictions of target values typically has numerous adjustable parameters. This could allow fitting the detail and noise in the training data to the extent that it negatively impacts the performance of the model on held-out data. In this case, the developed algorithm is of no use even if prediction accuracy is high as it cannot generalize over unseen samples. This problem is well known in the machine learning field under the name of “overfitting.” To verify that any increase in accuracy over the training subset is generalizable, an evaluation of the algorithm using a portion of unseen data (*validation subset*) should be done. It is essential that the validation subset does not contain samples presented in the training subset. However, during the design of training and validation subsets, one should note that genomic objects that are not equivalent from a mathematical point of view might share a large amount of biological information. For example, nested chromatin loops might share a large portion of epigenetic information encoded by the window spanning loop anchors although the anchors themselves do not overlap and formally represent different pairs of genomic regions. Such indirect overlapping results in the sharing of information between training and validation data sets, leading to the overestimation of prediction accuracy (Belokopytova et al., 2020). To overcome this problem, one can use different chromosomes for training and validation data sets.

It is considered that machine learning–based algorithms can find complex non-linear patterns when fitting the model. Machine learning is used for binary classifiers for regression-based models, enabling the prediction of structures ranging from two-point interactions to whole Hi-C maps. Several algorithms employing these methods for promoter–enhancer interaction prediction were recently developed, including TargetFinder (Whalen et al., 2016), DeepTACT (Li et al., 2019), 3DPredictor (Belokopytova et al., 2020), and HiC-Reg (Zhang et al., 2019). We refer the reader to the informative review of Xu et al. (2020) describing different algorithms for the prediction of enhancer-promoter interactions. Other spatial chromatin structures, such as loops (Zhu et al., 2016; Al Bkhetan and Plewczynski, 2018; Kai et al., 2018; Zhang et al., 2018; Trieu et al., 2020) and contact probabilities (Zhang et al., 2019; Belokopytova et al., 2020; Fudenberg et al., 2020; Schwessinger et al., 2020) also can be predicted by machine learning–based algorithms (see the section above). Furthermore, a machine learning–based approach enables revealing biological features underlying 3-D genome folding, which improves our understanding of biological mechanisms. For example, extracting matrix positional weights from layers of convolution neural networks helps to find the main features, in particular, sequences giving the main contribution to the prediction and consequently to the 3-D chromatin structure. Another example is the analysis of feature importance in a gradient-boosting algorithm that gives the ranked list of features that helps to find the best feature. Anyway, analysis of features and algorithm parameters can inspire thoughts of biological mechanisms underlying the studying process.

### Challenges and Limitations

#### Defining Target Features and Their Properties

The development of a predictive algorithm should start from a clear statement of biological features one wants to predict. Clear definitions of the features are important for the selection of positive and negative samples as well as for the choice of the machine learning algorithm.

Let us consider the goal of the prediction of interacting promoter–enhancer pairs. How would one define positive cases, i.e., interacting pairs? Now, it is clear that the majority of loops (see Box 1 for details of mechanisms underlying these structures) observed on Hi-C maps are due to the synergetic activity of cohesin and CTCF proteins. These complexes form loops that might facilitate interactions of promoters and enhancers located within the looping region by reducing the spatial distance between them but do not necessarily directly mediate contacts between these regulatory elements. In accord with this, direct functional tests based on targeted enhancer deletions or CRISPR-interference approaches (Gasperini et al., 2019) indicate that the vast majority of interacting enhancer–promoter pairs do not overlap with loop anchors although they are often located within a reasonable distance from them (Belokopytova et al., 2020). Thus, functionally interacting enhancer–promoter pairs might show only a slight increase in contact frequency. It is worth noting that the NG Capture-C approach (Davies et al., 2015) provides more sensitive and robust quantitation and enables detecting more significant interactions than Hi-C; however, typical Hi-C data are more widespread and available. At the same time, the majority of algorithms predicting 3-D genome structures are classifiers, so they solve the question of whether the promoter and enhancer interact, answering yes or no. We argue that quantitative measurement and prediction of spatial enhancer–promoter interactions are more informative than qualitative attribution to the loop anchors, and regression-based methods are more suited for such predictions.

Another example of varying feature definition is loop prediction. In this case, authors often use loops called by specific algorithms as positive samples. A large proportion of loop calls varies between algorithms and visually assessed loops (Belokopytova et al., 2020; Salameh et al., 2020). Methods for loop detection, such as for TAD detection, are constantly improving. For example, the last published method Peakachu for loop calling can detect more loops than previous algorithms (Salameh et al., 2020). The same applies to TAD calling: Zufferey et al. (2018) compared 22 different TAD caller algorithms and found that TAD sizes and numbers vary significantly among callers and data resolutions.

To sum up, it is very important to consider the nature and biological properties of target features and carefully design positive and negative samples if using classifiers for prediction.

#### Predicting Single-Cell Data

The statistical approach is well applicable for 3-D genome structure prediction and investigation, but it uses population data. It allows getting a prediction that is actually a mean value for a cell population, which does not provide information about the 3-D genome organization of a single cell and differences of spatial contacts between distinct cells. Conversely, physical modeling always produces ensembles of single-cell chromatin configurations. Nevertheless, it does not mean that this prediction matches a real biological cell exactly even if its average matches population Hi-C data. However, recently Conte et al. (2020) show the consistent agreement between the predicted structures and independent single-cell super-resolution microscopy data, which provides evidence that, at least in the studied loci, polymer physics approaches accurately capture single-cell chromatin conformation. This issue is under active debate, however.

#### Understanding Mechanisms Underlying Prediction

Another limitation is that one cannot extract a simple algebraic formula transforming features into target feature values from a trained machine learning model. Therefore, the statistical dependencies found by machine learning algorithms are difficult to interpret in biological terms. Nevertheless, it is possible to evaluate the feature’s contribution to prediction. We have already discussed several approaches for estimation of feature importance above; in addition, modifying features *in silico* and accessing how the modifications impact prediction could provide insights about the role of biological features used for prediction (Fudenberg et al., 2020).

#### Choosing Data Parameterization Function

To train a machine learning model, input data should be represented in a specific format, typically as a numeric vector of fixed length. The process of conversion of the input data into the desired format is called parameterization, and choosing the parameterization function might not be trivial. For example, ChIP-seq data is often used for the prediction of spatial chromatin contacts. There are several ways to submit these data to the algorithm: as a sum of ChIP-seq signals in the interval between two genome loci of interest, the total number of peaks in this region, the signal value of the nearest ChIP-seq peaks, or the *p*-values of peaks, etc. In our experience, differences in parameterization could significantly affect prediction accuracy. Thus, the most challenging part is to choose the best way of parameterization to achieve the best performance of the algorithm.

#### Input Data Quality

Another important issue is the quality of the training data. Some machine learning algorithms are sensitive to outliers presented in the data. In this case, data smoothing should be performed before training the model. For example, for Hi-C and RNA-seq data, it is often useful to log-transform values.

Recently, high-resolution Hi-C maps were published (Hsieh et al., 2015, 2020; Krietenstein et al., 2020). They reveal chromatin structures in more detail and thereby improve predictions. Moreover, we noticed that the prediction of higher resolution heat maps is more accurate than the prediction of the same heat map but with a lower resolution (Belokopytova et al., 2020). This aspect is explained by features used for prediction. We gain lots of information from ChIP-seq data, in which the protein-binding event is attributed to a small locus (usually less than 200 base pairs). In this case, using an ultra-high resolution of Hi-C maps provides a better correspondence between protein-binding sites and interacting loci, allowing the model to learn effects mediated by specific proteins in a more direct way.

#### Overfitting

Another problem of machine learning approaches is overfitting. In this case, the model performs well on the training data set but does not perform well on a holdout sample, actually not capturing real complex patterns underlying the 3-D genome structure. Non-overlapping subsets for training and validation help to detect overfitting. There are two main ways to minimize overfitting: training the network on more examples and changing the complexity of the network. However, in the case of biological data, it is not always possible to have enough training samples. To increase the number of samples, it may be necessary to combine data from multiple sources. This leads to the next challenge: to normalize data from different sources that require rigorous data preprocessing (Xu and Jackson, 2019).

## What Do We Consider a Good Prediction?

Any data type has its data specificities, and this is also true for the Hi-C maps discussed below. It should be remembered that, usually for 3-D chromatin architecture, prediction binary classifiers or regression-based methods are used. There are some common metrics to access the binary classifier’s performance, such as f1-score, AUC, and others. These metrics do not have any special characteristics related to genomic data.

The performance estimation of regression-based methods is more specific for Hi-C maps. How can we understand that one heat map is similar to another? Actually, a Hi-C map is a matrix of numbers, so we can apply any metrics for matrices comparison.

**FIGURE 3:**
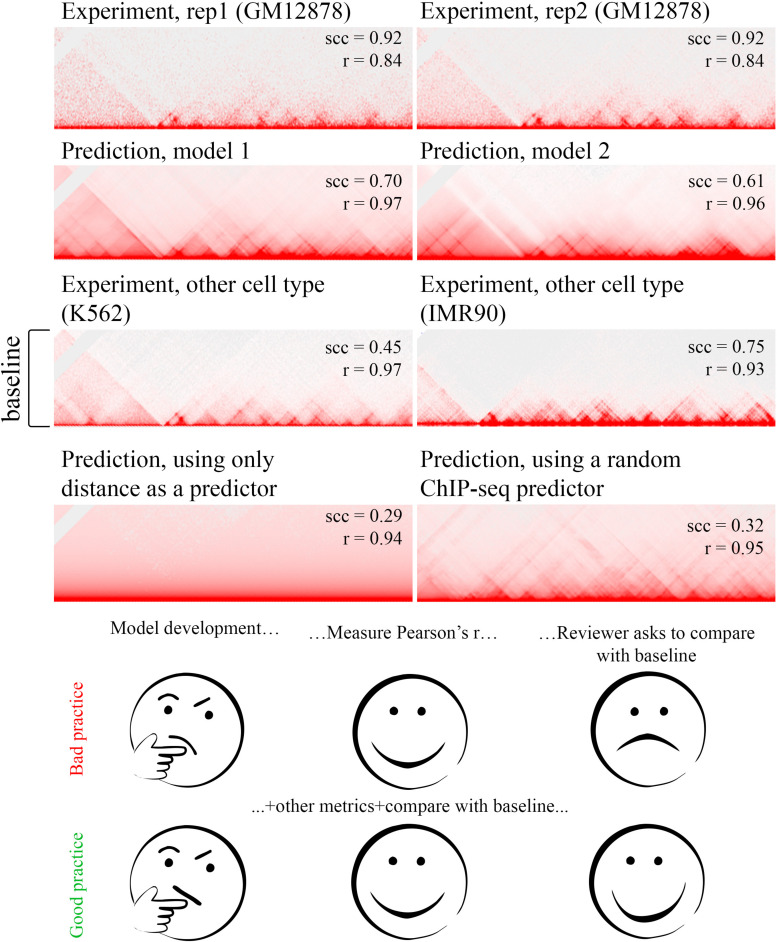
The choice of the baseline plays a key role in assessing the prediction accuracy. Experimental data are from Rao et al. (2014); predictions generated using 3DPredictor (Belokopytova et al., 2020) supplemented with following data: genomic distance, CTCF, and RNA-seq (model 1) (Qi and Zhang, 2019) (model 2).

The basic metric is Pearson’s correlation. Let us consider, for instance, a Pearson’s correlation equal to 0.8: Does this correspond to a good or bad prediction? Intuitively, it seems that a Pearson’s correlation equal to 0.8 indicates accurate prediction. However, using absolute values is not a good idea. As we discussed above, contact probability shows prominent dependence from distance, and even very simple prediction algorithms efficiently capture this dependence. Even when the distance between loci is not directly provided, it could be inferred from many epigenetic features. For example, cumulative ChIP-seq signals scale with the length of the genomic region, allowing prediction of contact probability. As we show in Figure 3, using randomly shuffled ChIP-seq signals, which have no biological meaning, allows the generation of predictions highly correlating with experimental data. Also, the whole-map correlation coefficient does not reflect the prediction of specific topological structures, such as TADs, loops, or compartments.

There are several workarounds allowing the comparison of Hi-C maps using correlation coefficients. First, one can compare the correlation between predicted and experimental data with the correlation between experimental replicates. Ideally, the prediction should be as similar to the experimental data as replicates among themselves. However, replicates are not always available; in addition, Tao Yang et al. show that Pearson’s correlation between unrelated samples sometimes is equal to differences between replicates (Figure 3 in Yang et al., 2017).

Another baseline could be obtained by scoring differences of Hi-C maps between distinct cell types. Chromatin organization is moderately conserved between different cell types (Dixon et al., 2012; Battulin et al., 2015) and even between different species (Fishman et al., 2019; Nuriddinov and Fishman, 2019), thus predicting cell type–specific features might be more challenging than an overall 3-D organization. For a high-quality algorithm, one would expect the difference between prediction and experimental data on the target cell type to be less than between different cell types. Besides this, one should carefully select data sets for comparison, accounting for their noise level. The lower noise level in the experimental data on target cell type results in higher measures of prediction accuracy, whereas a high noise level in a cell type used for baseline results in low baseline metrics, thus overestimating predictive power.

To overcome the limitations of standard correlations as measurements of Hi-C map similarity, Tao Yang et al. propose a framework that minimizes the effect of noise and biases by smoothing the Hi-C matrix, and then it addresses the distance-dependence effect by stratifying Hi-C data according to their genomic distance (Yang et al., 2017). This SCC metric distinguishes subtle differences between closely related cell lines, biological replicates, and pseudoreplicates, which was shown in the paper (Figure 3 in Yang et al., 2017).

Besides Pearson’s correlation and SCC standard metrics for comparison of matrices, such as MAE, MRE and others can be used for algorithm performance estimation. Similar to Pearson’s correlation, understanding the values of these metrics requires a comparison with the baseline. Overall, we recommend using several metrics and several baselines for the optimal assessment of prediction accuracy (Figure 3).

Nevertheless, it is useful to visualize the predicted Hi-C map for empirical assessment to be confident that the chosen metric correctly reflects the differences between heat maps. Another way is to estimate the prediction of 3-D chromatin structures, such as TADs and loops. For some statistics, one can call loops or insulator boundaries at experimental and predicting maps and then compare and overlap detected structures.

The selection of metrics for prediction accuracy estimation is an important issue for every algorithm. It should correctly reflect differences of 3-D chromatin features.

## Prediction of Functional Consequences of Rearrangements

Some rearrangements have been known to change the 3-D chromatin structure, causing diseases. Several works show the importance of chromatin folding in the gene regulation process (Franke et al., 2016; Rodríguez-Carballo et al., 2017; Kraft et al., 2019). Inversions, duplications, and other rearrangements can lead to TAD disruption, changing of promoter–enhancer interactions, and the emergence of new interactions between regulatory elements and genes. These insights are significant for medical genetics because the interpretation of chromosomal rearrangements in non-coding regions remains a big challenge. Zepeda-Mendoza et al. (2018) suggest detailed instructions on how to run a computational pipeline that identifies relevant candidates of non-coding balanced and apparently balanced chromosomal abnormality position effects. This pipeline includes analysis of TADs and the possibility of changing enhancer–promoter interactions due to rearrangement. Hence, the analysis of chromosomal rearrangement consequences in the context of the 3-D genome structure becomes a routine assay. The recently published machine learning algorithm TADA (Hertzberg et al., 2020) can prioritize large chromosomal alterations, such as copy number variants (CNVs) based on their pathogenicity.

Besides the prediction of the overall rearrangement effect, it is possible to predict changes in 3-D genome structures as TADs and loops. The 3D-GNOME algorithm (Sadowski et al., 2019; Wlasnowolski et al., 2020) generates chromatin 3-D structures using a Monte Carlo approach based on chromatin conformation capture (3C) data. It uses high-quality CTCF or RNA polymerase II ChIA-PET data as a reference chromatin interaction pattern. For rearrangement prediction, it applies a series of simple rules to recover chromatin interaction patterns. The 3D-GNOME algorithm can visualize alterations emerging in genomic structures after the introduction of SVs^1^. Another approach is to predict changes in chromatin loops by a machine learning–based DeepMilo algorithm (Trieu et al., 2020). The algorithm can extract features directly from DNA sequences of loop anchors not using information about the presence and orientation of CTCF motifs. It allows predicting true Hi-C loops not having a CTCF signal at their anchors. DeepMILO can predict effects even of small mutations, and authors identified insulator loops predicted to change in multiple cancer patients and genes affected by these loops.

The aforementioned algorithms predict the perturbation of specific chromatin structures, such as loops and TADs. Other tools are capable of predicting a complete Hi-C map of the mutated locus. Algorithms such as Akita (Fudenberg et al., 2020), DeepC (Schwessinger et al., 2020), 3DPredictor (Belokopytova et al., 2020), PRISMR (Bianco et al., 2018), and others can predict alterations of 3-D chromatin architecture induced by structural variants.

An area of increasing interest and active research is the effect of small INDELs and single base pair variants on chromatin architecture. It is known that even single nucleotide replacement can lead to changes in 3-D genome structure, for example, by modifying CTCF binding sites (Schmiedel et al., 2016; Sun et al., 2020). A separate mission of predictive algorithms is to foresee the consequences of such mutations. Some algorithms, such as DeepMILO (Trieu et al., 2020), Akita (Fudenberg et al., 2020), and DeepC (Schwessinger et al., 2020) use a nucleotide sequence as the main feature for prediction. These algorithms are very powerful in predicting changes induced by small mutations because the mutations directly affect input features. On the other hand, training these algorithms requires knowledge of 3-D chromatin organization in wild-type cells of the same type because a nucleotide sequence does not provide cell type–specific epigenetic information.

Other algorithms do not use nucleotide sequences for prediction directly. In this case, it is important to model changes in input features caused by SNP or small INDEL. For instance, in the case of polymer modeling, it needs to change binder position or to remove the part of the polymer corresponding to the mutated DNA. All the same is about statistical approaches not using nucleotides as features for the prediction.

## Conclusion

The mechanisms that underlie genome organization are intensively studied. Multiple groups developed computational algorithms to explain mechanisms underlying genome architecture and predict chromatin folding in normal and mutated cells. However, there is still no approach that is able to completely describe the whole complexity of the nuclear organization. Physical models are limited by incomplete knowledge of mechanisms and relevant system parameters, such as interaction affinities and concentrations. Statistical methods do not allow understanding of the exact mechanisms underlying captured dependencies. And for both methods, it is not clear whether developed algorithms trained and validated using several cell types could be broadly and efficiently transferred to other cell types and conditions.

The latter question could be answered using the rapidly growing number of high-resolution Hi-C data sets. There are multiple published experimental data studying 3-D genome structure in normal and rearranged genomes. Such experiments provide detailed Hi-C maps of mutated regions that can be used as validation data for predictive algorithms. We believe that benchmarking and comparing existing predictive algorithms using these data sets would help to describe their power and limitations and to develop new, comprehensive approaches for the prediction of chromatin organization and dynamics in the future.

## Author Contributions

Both authors listed have made a substantial, direct and intellectual contribution to the work, and approved it for publication.

## Conflict of Interest

The authors declare that the research was conducted in the absence of any commercial or financial relationships that could be construed as a potential conflict of interest.

## Footnotes

1 https://3dgnome.cent.uw.edu.pl/
